# Spontaneous Combustion

**Published:** 1851-09

**Authors:** 


*Spontaneous Combustion.* — The question cf spontaneous combustion being involved in a trial, recently, at the capital of the Grand Duchy of Hesse Darmstadt, and scientific opinions on the point conflicting with each other, the Judge directed Profs. S. Bischoff, and J. De Liebig, to be summoned, in order, by their investigations and testimony, to resolve doubts as to the probability of the occurrence of such an event.

A report of the case appeared in the *Archives Generales de Medicine*, Paris, which has been translated by a correspondent of the Western Journal of Med. and Surgery. The details of the case are interesting, but we are obliged to omit them on account of the space which they would occupy.

We give the oral report of these distinguished Professors on the subject of spontaneous combustion, aside from the particular facts in the trial referred to. It will be seen that they are convinced of the impossibility of such an occurrence.—*Editor Buffalo Med. Journal.*

The first recorded case of spontaneous combustion, said Mr. Bischoff, dates back about one hundred and fifty years (that of Millet a women of Reims, in 1725.) By human *spontaneous combustion*, we understand that a man has been more or less burned, without our being able by external circumstances to explain the burning. Then it is that we say, this man has burned not by aid of external combustibles, but within himself (spontaneously;) an expression, which, implying an entire theory, seems to be incorrect. How much better it would be to say: we do not know how this person died, but, in saying that he died from spontaneous combustion, we only substitute an explanation quite as absurd. Thus the doctrine of spontaneous combustion, which has crept into the science, is the result of ignorance, and not of scientific research or experimentation. Upon what authorities does this pretended fact rest? The forty-five or forty-eight cases which have been published, relate to individuals burnt either where there was too little, or an entire absence of, combustible material, to account for the combustion. With the exception of two cases of complete and four of partial consumption by fire, the victims of which survived, no one possessing scientific knowledge, no witness of any sort, has been present during the accident. The first case of complete spontaneous combustion, was witnessed and described by a chamber-maid and an unknown person, and the second by a stranger. In the four cases of partial combustion, it is very evident that the first one was caused by burning sulphur, which the burnt person spread upon his clothing in the endeavor to extinguish the flames with his hands; the second was the case of a young girl of Hamburg, upon whose hands there appeared a flame which was followed by vesicles; but this fact though reported by a distinguished physician, was not witnessed by himself, or by any other person. Most probably then, these vesicles were the result of some disease, or perhaps of a burn intentionally made, for the purpose of attracting attention, of gaining admission into the hospital. The third case, which is very generally known, relates to a priest, named Bertoli, and was reported in 1786, by an Italian surgeon. Any unprejudiced person, who reads the account of this case, must be convinced that that individual was burnt by fire communicated to his clothing from a lamp. The fourth is still less worthy of confidence; it relates to a blacksmith named Reynaleau: a narration unconfirmed by the slightest evidence, full of contradictions, and winding up with a characteristic trait, that holy water only was able to extinguish the flames. Not one of these cases has ever been reported immediately after its occurrence by any one of recognized authority. Physicians have arrived in several instances after the catastrophe; and some of the cases have been subjected to a judicial inquest, and have the appearance of reality. But for an observer accustomed to criticism, and to the requirements of rigid observation, the reports which we possess are far from presenting the guaranties indispensable to an enlightened investigation. Phenomena such as those of spontaneous combustion, require a methodical observation, which the cases mentioned do not possess the slightest trace of. In none of them was there even an autopsy made, still less a serious, scientific investigation, or chemical analysis. In all the cases of spontaneous combustion which have called forth judicial or medical inquests, we constantly see an exhibition of levity, ignorance, prejudice, credulity, and very often of culpability. At the epoch when most of these facts were reported, science itself was not sufficiently far advanced to supply the lights necessary for an analysis of what was observed. As to the numerous cases that have been reported from hearsay, they are based upon the authority of the school-master, the curate, or the village mayor, but have never given rise to an inquest of any kind. A recent and very remarkable instance will demonstrate how, gradually, these accounts become introduced into the annals of the science. M. Bischoff here cited the case of pretended spontaneous combustion, published by the *Gazette des Tribunaux*, and reproduced in the *Journal des Debats*, for February 24th, 1850. The case was that of a man who, in a drinking shop, where he had, according to custom, drank deeply, introduced into his mouth in consequence of a bet, a lighted candle; he was suddenly set on fire from within, and his head and the upper part of his chest were carbonized in half an hour in spite of all the assistance rendered. The death and the effects of the fire were reported to have been verified by two physicians. From information acquired by M. Liebig from different savans of Paris, and especially from the prefect of police, it turns out that the whole account was imaginative, and pure fiction, invented for the columns of the paper which had inserted it.

The question of spontaneous combustion has been treated of, adds M. Bischoff, by Rudolphi and Treviranus, MM. Kopp and Nasse. These savans have investigated and have expended a great deal of labor and of science for the purpose of explaining this phenomenon. But incredible as it may seem, without first assuring themselves of the truth of reports as made, they have admitted them as they were presented. Their explanation only could be important, and this is wanting. We do not deny these cases because we cannot explain them, but because their existence must be based upon explanations which tend to overthrow the laws, heretofore admitted as true and exact, of physics, physiology, chemistry and pathology. I will confine myself to the mention of the fact, recognized by all, that a body containing twenty-five per cent. water, does not take fire of itself, and does not continue to burn when started. Suppose we collect all the solid parts of the body, the bones, the skin, the tendons, the muscles, and put the water contained in the body into a vase; kindle these solid parts and their entire consumption will not afford sufficient heat to vaporize the water. Alcoholic excess, however, we are told, brings about a modification in the human body which renders possible its spontaneous combustion. It is true, all accounts of cases of spontaneous combustion tell us that the subjects were addicted to the abuse of ardent spirits; it is also true that alcohol is inflammable; and why not admit that the body soon becomes impregnated with it, and that, especially when fire is communicated from the exterior, it can burn. Such reflections may be made, but for the naturalist and physician they are valueless. The knowledge which we possess upon the passage of substances from the stomach and intestines into the blood, teaches us that alcohol reaches slowly, and in very small quantities, the sanguineous system, and as the circulation goes on with great rapidity, the alcohol is carried almost immediately into the lungs, where the blood comes in contact with the air. There the elements of the alcohol become modified by its combination with the oxygen of the air, forming carbonic acid and water which the respiratory process eliminates from the system. Thus under ordinary circumstances we cannot even prove the presence of alcohol in the blood, since it is decomposed and thrown off by the lungs. Here it may be thought that alcohol taken abundantly will penetrate the blood in substance and in large quantities, and spread itself throughout the whole system. Observations and experiments by distinguished men have been made upon this subject, but they are contradictory; some pretend to have found alcohol in the blood, and even in the brain of persons addicted to drink and who have died drunk. Percy discovered traces of it in the brains of dogs into whose veins he had injected a considerable dose. But the observations of Percy are in direct contradiction to those of Dr. De Pommer, of Zurich, who found no traces of the spirit in the blood. MM. Bouchardt and Sandras were unable to discover alcohol in any secretion except in the pulmonary exhalation. So far then it remains to be proved that the human body can imbibe alcohol like a sponge; moreover it is, *a priori*, impossible to admit that the body being saturated with alcohol life could continue for a single instant. The coagulation of the albumen, the arrest of the circulation, and the destruction of the nervous system, are the immediate results of the injection of any considerable quantity of alcohol into the blood of an animal. For my own part I am convinced that a dead body does not become combustible from being saturated with alcohol. I have taken parts of a dog into whose arteries I had injected alcohol at 92°, and they would not burn when exposed either to a flame or to the action of carbon; in the latter case only they roasted and carbonized, but ceased even that so soon as withdrawn from the action of the fire. M. Bischoff ended by refuting and ridiculing the stories of flames issuing from the mouths of drunken individuals.

M. Liebig, who, six years since, had given his opinion upon spontaneous combustion, (*Annales de Chimie et de Physique*, 1844, t. 1, page 331,) likewise opposed, for very extensive reasons, the possibility of the facts reported on the subject. It will be borne in mind, said he, that the idea of spontaneous combustion arose at a period when all opinions upon the subject were erroneous. What takes place in combustion was only known some seventy years ago, (Lavoisier.) What is necessary for the combustion of a body, has been known only forty years, (Davy.)

Since the occurrence of the case of Millet of Rheims to the present time, some forty-five or forty-eight cases have occurred which are alike in — 1st. Always having taken place during the winter season; 2d. The persons attacked have all been drunkards, drunk at the time; 3d. They have most generally happened in countries where rooms are heated by open fire-places, and furnaces of charcoal, in England, France, and Italy; in Russia and Germany, where stoves are principally used, deaths from spontaneous combustion are very rare; 4th. There have been no eye-witnesses to the combustion; 5th. No physician, amongst all those who have attempted to explain these cases, has seen one; 6th. There is no information as to the quantity of combustible matter consumed; and 7th. Some time has always elapsed from the commencement of the combustion until the body has been found consumed. M. Liebig also argued with great power upon the principal details reported as connected with spontaneous combustion, and upon the explanations given of them. He also demonstrated clearly the error into which the partizans of the spontaneous combustion theory have fallen. The arguments which they employ being deduced, contrary to all logical rules, in the cases in point; death and the destruction of the body, the cause of which is unknown, being assumed as proof of the truth of the assumed cause. These cases are explained by the fact of the possibility of spontaneous combustion, the existence or possibility of which is proved by the same cases. This discussion we are disposed to believe does not admit of refutation, and gives a fatal blow to the doctrine of spontaneous combustion.

The trial, which occupied the greater part of the month of March, 1850, concluded by the condemnation of J. Strauss, as the murderer of the Countess of Goerlitz, to perpetual imprisonment. The journals have recently published that he has made a full acknowledgment of his crime. He admitted that going into the chamber of the Countess upon some household duty, and finding no one, he could not, upon seeing that the secretary contained money and articles of value, resist the temptation to steal. The Countess having discovered him in the act, he seized and strangled her with some difficulty; then having placed the body in an arm chair, near the secretary, he surrounded it with combustibles, to which he set fire for the purpose of concealing his crime.

